# Assessing the bipotency of in vitro-derived neuromesodermal progenitors

**DOI:** 10.12688/f1000research.6345.2

**Published:** 2015-07-31

**Authors:** Anestis Tsakiridis, Valerie Wilson

**Affiliations:** 1 MRC Centre for Regenerative Medicine, Institute for Stem Cell Research, School of Biological Sciences, University of Edinburgh, Edinburgh, EH16 4UU, UK

**Keywords:** Neuromesodermal progenitors, Axis elongation, Pluripotent stem cells, Paraxial mesoderm, Neurectoderm, In vitro differentiation, Primitive streak

## Abstract

Retrospective clonal analysis in the mouse has demonstrated that the posterior spinal cord neurectoderm and paraxial mesoderm share a common bipotent progenitor. These neuromesodermal progenitors (NMPs) are the source of new axial structures during embryonic rostrocaudal axis elongation and are marked by the simultaneous co-expression of the transcription factors T(Brachyury) (T(Bra)) and Sox2. NMP-like cells have recently been derived from pluripotent stem cells *in vitro* following combined stimulation of Wnt and fibroblast growth factor (FGF) signaling. Under these conditions the majority of cultures consist of T(Bra)/Sox2 co-expressing cells after 48-72 hours of differentiation. Although the capacity of these cells to generate posterior neural and paraxial mesoderm derivatives has been demonstrated at the population level, it is unknown whether a single *in vitro*-derived NMP can give rise to both neural and mesodermal cells. Here we demonstrate that T(Bra) positive cells obtained from mouse epiblast stem cells (EpiSCs) after culture in NMP-inducing conditions can generate both neural and mesodermal clones. This finding suggests that, similar to their embryonic counterparts, *in vitro*-derived NMPs are truly bipotent and can thus be exploited as a model for studying the molecular basis of developmental cell fate decisions.

## Introduction

Axis elongation in vertebrate embryos proceeds in a rostral-to-caudal sequence and involves the coordinated production of spinal cord neurectoderm and paraxial mesoderm/somites from a population of neuromesodermal progenitors (NMPs) (for a review see 1). The bipotent status of these axial stem cells was demonstrated in the mouse by retrospective clonal analysis ^2^. NM-potent cells are located in the node-streak border and the adjacent caudal lateral epiblast of early somite stage embryos and in the chordoneural hinge (CNH) region of the tail bud of later stage embryos ^3– 5^ i.e. in areas exhibiting high levels of Wnt and FGF signaling ^1^. The main hallmark of these cells is the co-expression of the mesodermal transcription factor T (Bra) together with the neural marker Sox2 ^6– 9^. NMPs are not only an excellent model for deciphering the mechanisms controlling cell fate choice (neuroectoderm vs mesoderm), but also comprise an attractive source for generating trunk spinal cord neurectoderm cells and skeletal muscle *in vitro*.

We and others have recently shown that mouse and human pluripotent stem cells cultured for 48–72 hours in the presence of FGF2 and the Wnt signaling agonist CHIRON99021 (CHIR) yield a high percentage of T(Bra) ^+^Sox2 ^+^ double-positive cells that transcriptionally resemble embryonic NMPs ^10, 11^. These NMP-like cells were also shown to efficiently differentiate exclusively into paraxial mesoderm and posterior neurectoderm both *in vitro* and *in vivo* upon grafting into cultured mouse and chick embryos ^10^ suggesting an NM bipotent character. However, these studies were carried out at the population level and it would thus be important to test the NM potency of single cells. Here we address this issue by showing, through the clonal plating of T(Bra) ^+^ cells generated after culture of epiblast stem cells (EpiSCs) ^12, 13^ in NMP-inducing conditions, that individual *in vitro*-derived NMPs are truly bipotent as they give rise to colonies consisting of both neural and mesodermal cells.

## Methods

### Cell culture, differentiation and flow cytometry

T(Bra)-green fluorescent protein (GFP) reporter (TGFP) EpiSCs were derived from TGFP embryonic stem (ES) cells (sourced from 14) and cultured routinely in fibronectin-treated plates in N2B27 medium containing 10 ng/ml FGF2 (R&D Systems) and 20 ng/ml Activin A (Peprotech) as previously described in 15. For NMP differentiation TGFP EpiSCs were plated at a density of approximately 1500–2000/cm ^2^ in N2B27 medium containing 20 ng/ml FGF2 and 3 µM CHIRON99021 (Stemgent) on fibronectin for 48–72 hrs ^10^. For clonal plating experiments *in vitro*-derived NMPs were pre-treated with 10 µM ROCK inhibitor Y-27632 (Calbiochem) for 1 hr prior to fluorescence-activated cell sorting (FACS). After this they were re-plated at a density of 4,000 cells/well in 12-well plates in medium containing either FGF2, or FGF2/CHIR alongside Y-27632 for the first 8 hours. We have previously found that when 1:1 mixtures of GFP ^+^ and GFP ^-^ EpiSCs are plated at a total of 5,000 cells/well in 12-well plates (or 10,000 cells/well in 6-well plates) then 95% of the resulting colonies between 2–8 cells are of monoclonal origin. Here we also included for scoring colonies of up to 10 cells since we employ a smaller initial plating density (4,000 cells/well) ^6^. For non-clonal plating of *in vitro*-derived NMPs, approximately 40,000 cells/well (12-well plate) were used. Cell sorting was performed using a FACSAria (BD Biosciences).

### Immunocytochemistry and microscopy

For immunocytochemistry cells were fixed with 4% paraformaldehyde, washed with PBS/0.1% Triton X-100 (PBST), treated with 0.5 M Glycine and blocked in PBST/3% donkey serum/7.5% bovine serum albumin (BSA). Primary antibody incubations were performed overnight at 4°C, followed by PBST washes the following day, incubation with secondary donkey Alexafluor antibodies (Life Technologies) for 2–3 hrs at room temperature and further washes in PBST. The primary antibodies used were: donkey polyclonal anti-T(Bra), 1 μg/ml (RRID: R&D Systems Cat# AF2085 RRID:AB_2200235), rabbit monoclonal anti-Sox2, 0.5 μg/ml (RRID: Abcam Cat# ab92494 RRID:AB_10585428) and goat polyclonal anti-Tbx6, 0.5 μg/ml (RRID: R&D Systems Cat# AF4744 RRID:AB_2200834). Fluorescent images were captured using an Olympus IX51 inverted microscope (Olympus) using a x20 objective and the Volocity software (PerkinElmer). Nuclear segmentation followed by single cell fluorescence quantification was performed as described previously ^16^. T(Bra) and Sox2 protein positivity scoring of individual clones was carried out manually.

## Results

**Figure 1.**
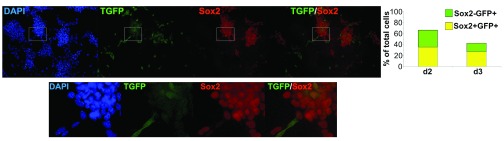
**Left**: Fluorescence analysis of TGFP and Sox2 expression in TGFP EpiSCs cultured for 48 hours in FGF2/CHIR following antibody staining against Sox2. **Right**: Quantification of TGFP ^+^Sox2 ^+^ and TGFP ^+^Sox2 ^-^ expressing cells in TGFP EpiSCs differentiated in NMP-inducing conditions after 2 (d2) or 3 (d3) days following immunocytochemistry and image analysis. Magnified versions of the areas marked by a white box are shown in the bottom panel.

To track the emergence of NMPs *in vitro* we employed a T(Bra) reporter EpiSC line (TGFP) generated from ES cells carrying a GFP transgene knocked into the T(Bra) locus ^14^. This reporter line has been shown to faithfully recapitulate endogenous T(Bra) expression. In line with our previous findings ^10^, culture of TGFP EpiSCs in the presence of FGF2/CHIR for 48 or 72 hours gave rise to a significant number of TGFP ^+^ cells, many of which were also positive for Sox2 expression (55% of the total TGFP ^+^ population at 48 hours and 65% at 72 hours) as revealed by antibody staining and image analysis ( Figure 1). Interestingly, TGFP ^+^Sox2 ^+^ cells appeared in “patches” and not in a “salt and pepper” manner, possibly reflecting our previous findings on the mutually exclusive emergence of distinct mesodermal precursors from a heterogeneous starting EpiSC population (6) or non-synchronous generation of NMP-like cells *in vitro*. In summary, these results indicate that at least half of the TGFP ^+^ cells emerging in the presence of FGF2/CHIR are NMP-like and thus we used TGFP expression under these conditions to enrich for cells with NMP identity.

**Figure 2.**
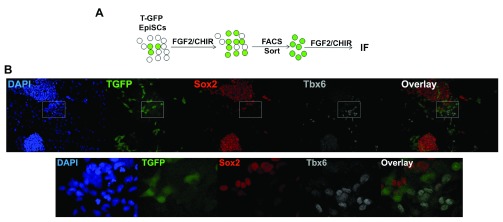
( **A**) Scheme depicting the differentiation and re-plating of *in vitro* induced NMPs at high density after flow sorting. ( **B**) Fluorescence analysis and immunocytochemistry of TGFP, Sox2 and Tbx6 expression of *in vitro*-derived NMPs sorted at day 2 of differentiation and re-plated at high density in the presence of FGF2/CHIR for 2 days. In all cases cell nuclei were visualized using DAPI. IF: immunofluorescence. Magnified versions of the areas marked by a white box are shown in the bottom panel.

We have previously found that prolonged (i.e. more than 72 hours) culture in FGF2/CHIR mediates further differentiation of NMPs into mutually exclusive paraxial mesoderm and neurectoderm cells ^10^. Therefore apart from promoting an NMP state these conditions simultaneously provide an environment for the production of the natural differentiation products of NMPs. We thus utilized culture in FGF2/CHIR in order to test the NM potency of TGFP ^+^ NMPs at the population level. TGFP EpiSCs were cultured in NMP-promoting conditions for 48 hours and the resulting GFP ^+^ cells were sorted by flow cytometry and re-plated at high density for a further 48–72 hours in the presence of FGF2/CHIR ( Figure 2A). We have previously shown that under these conditions hardly any pluripotent cells persist in the differentiating cultures as evidenced by analysis of Nanog/Oct4 expression and grafting into the pluripotency-permissive environment of cultured E7.5 embryos ^10^. Immunofluorescence analysis of the final cultures showed that sorted TGFP ^+^ cells generated predominantly mutually exclusive single T(Bra) positive mesoderm and single Sox2 ^+^ neurectoderm ( Figure 2B). The cultures also contained clusters of Tbx6 ^+^ cells which were distinct from the T(Bra) ^+^ and Sox2 ^+^ domains ( Figure 2B) and, since this gene uniquely marks emergent paraxial mesoderm, these cells probably arose from the T(Bra)-expressing population. Together these data confirm that the TGFP-expressing cells produced in NMP inducing conditions possess the ability to generate both neural and mesodermal cells upon further differentiation.

**Figure 3.**
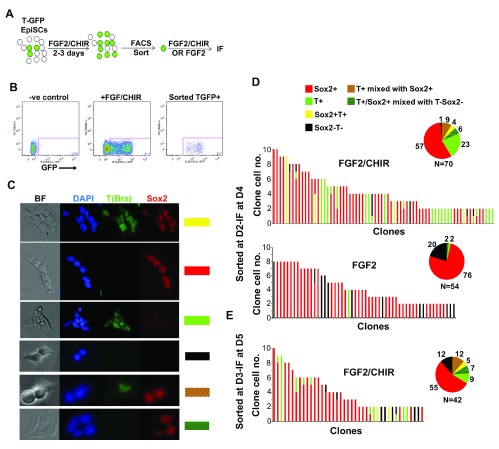
( **A**) Scheme depicting the differentiation and re-plating of *in vitro* induced NMPs at clonal density after flow sorting. ( **B**) FACS plots depicting analysis of TGFP expression in day 3 FGF2/CHIR-treated TGFP EpiSCs (middle). The purity of the GFP ^+^ sorted population and a negative control (wild-type EpiSCs) are also shown. ( **C**) Representative examples of the clones obtained after culture of single sorted TGFP ^+^ NMPs in FGF2/CHIR medium following immunofluorescence analysis of T(Bra) and Sox2 expression. The colour-coded bars on the right correspond to the scoring groups shown at the top of panel 3D. ( **D**– **E**) Composition of colonies obtained after clonal plating of TGFP ^+^ NMPs sorted at day 2 ( **D**) or day 3 ( **E**) for a further 48 hrs in FGF2/CHIR or FGF2 only. The cell number of each clone (in x axis) is shown in the y axis. Colour codes: Sox2+: single Sox2+ cells; T+: single T+ cells; Sox2+T+: Double positive cells; Sox2-T-cells: double negative cells; T+ mixed with Sox2+: clones consisting of single T(Bra)+ cells mixed with single Sox2+ cells; T+/Sox2+ mixed with T-Sox2-: clones consisting of single T(Bra)+ or single Sox2+ cells mixed with double negative cells. Pie charts: percentages of different classes of clones representing groups of the colour-coded phenotypes described above. Total numbers of clones scored are shown below each pie chart.

We next examined the behaviour of TGFP ^+^ NMPs at the single cell level. TFGP ^+^ cells induced after 48 or 72 hrs of FGF2/CHIR treatment were flow sorted (purity >99%) and re-plated at clonal density in FGF2/CHIR-containing medium ( Figure 3A,B). After 48 hours the resulting colonies were analysed by immunofluorescence and categorized based on their composition ( Figure 3C). Strikingly, most (55–60% of total) clones obtained from both day 2 and day 3 FGF2/CHIR-induced TGFP ^+^ cells were composed exclusively of single Sox2 ^+^ neurectodermal cells indicating a strong neurogenic capacity ( Figure 3D,E). The proportion of single Sox2 ^+^ colonies was significantly enhanced to 76% (p value<0.05 based on a two-tailed z test) with a concomitant decrease in the proportion of T(Bra) ^+^ cells when isolated single TGFP ^+^ cells produced after 2 days in FGF2/CHIR medium were re-plated in the presence of FGF2 alone for 48 hrs prior to clone scoring ( Figure 3D) confirming the pro-mesodermal effect of Wnt activity on NMPs ^8, 10^. We also observed purely mesodermal clones consisting of T(Bra) ^+^ cells which were particularly prominent in the case of sorted day two TGFP ^+^ NMPs ( Figure 3D,E). These data suggest that many *in vitro*-derived NMP cells are biased by the signaling environment towards unilinear differentiation into either neurectoderm or mesoderm. However, we did observe clones which comprised combinations of single positive T(Bra) ^+^ and Sox2 ^+^ cells (9% for day 2 and 12% for day 3 TGFP ^+^ NMPs) and were thus indicative of neuromesodermal potency. A few clones were found to contain only T(Bra) ^+^Sox2 ^+^ double positive cells ( Figure 3D,E) possibly reflecting NMP self-renewal. Finally, a small number of colonies were composed only of T(Bra) ^-^Sox2 ^-^ negative cells ( Figure 3D,E) which may represent more differentiated NMP derivatives such as spinal cord cells or mesodermal lineages other than paraxial (e.g. lateral/ventral) derived from sorted TGFP ^+^Sox2 ^-^ cells. Interestingly, we detected no Tbx6 ^+^ cells present in the clones (Representative, raw images shown in Dataset 5) despite their presence in cultures derived from sorted day 2 FGF2/CHIR-induced TGFP ^+^ cells plated at high density under the same conditions. This suggests that the presumed maturation of T(Bra) ^+^ cells into Tbx6-positive paraxial mesoderm depends on paracrine (e.g. FGF) ^17^ or juxtacrine (e.g. Notch) ^18^ signaling effects which are absent from the low density, clonally-derived cultures.

Supplementary data for Dataset 1 - Figure 1: Raw immunocytochemistry images. Legend: Fluorescence analysis of TGFP and Sox2 expression in TGFP EpiSCs cultured for 48 hours in FGF2/CHIR following antibody staining against Sox2.
Dataset 2 - Figure 2: Raw immunocytochemistry images. Legend: Fluorescence analysis and immunocytochemistry of TGFP, Sox2 and Tbx6 expression of in vitro-derived NMPs sorted at day 2 of differentiation and re-plated at high density in the presence of FGF2/CHIR for 2 days.
Dataset 3. Figure 3B - FACS data. Legend: FACS plots depicting analysis of TGFP expression in day 3 FGF2/CHIR-treated TGFP EpiSCs.
Dataset 4. Figure 3C - Raw immunocytochemistry images. Legend: Representative examples of the clones obtained after culture of single sorted TGFP+ NMPs in FGF2/CHIR medium following immunofluorescence analysis of T(Bra) and Sox2 expression (Images in figure 3C are magnified parts of raw images).
Dataset 5. Raw data - Tbx6-negative cells. Legend: Tbx6-negative cells in a clonal population of day 3 TGFP+ NMPs. Clones obtained after culture of single sorted TGFP+ NMPs in FGF2/CHIR medium following immunofluorescence analysis of T(Bra) and Sox2 expression. Click here for additional data file. Copyright: © 2015 Tsakiridis A and Wilson V 2015 Data associated with the article are available under the terms of the Creative Commons Zero "No rights reserved" data waiver (CC0 1.0 Public domain dedication).

## Discussion

The production of axial tissues during embryonic elongation is driven by posteriorly-located progenitors emerging round the end of gastrulation. A long-standing question in the field has been whether this cell population represents a mixture of separate unipotent neural and mesoderm-committed precursors or consists of bipotent progenitors. Genetic marking of single cells and their derivatives using the LaacZ system in mouse embryos shed light on this problem by revealing that spinal cord neurectoderm and paraxial mesoderm originate from bipotent neuromesodermal progenitors ^2^. These NMPs have also recently been captured *in vitro* through the culture of pluripotent stem cells in Wnt and FGF signaling agonists ^10, 11^. However, the bipotent status of these cells had not been previously demonstrated at the clonal level. Here we show that single *in vitro*-derived NMPs can give rise to mixed clones containing both neural (Sox2 ^+^T(Bra) ^-^) and mesodermal (Sox2 ^-^T(Bra) ^+^) cells, a finding which indicates that FGF2/CHIR-induced cultures contain *bona fide* NM bipotent cells.

Interestingly, a considerable fraction of individual sorted NMPs produced exclusively neurectodermal or mesodermal clones suggesting that a proportion of the Sox2 ^+^T(Bra) ^+^ cells induced from EpiSCs after 2–3 days in the presence of FGF2/CHIR may already be biased towards adopting a neural or mesodermal fate under conditions promoting both lineages. This may be a reflection of heterogeneity in the relative levels of Sox2 and T(Bra) protein/transcript within the *in vitro*-derived Sox2 ^+^T(Bra) ^+^ population with double-positive cells exhibiting higher levels of Sox2 showing a pro-neural bias while T(Bra) ^High^ cells are predisposed to mesoderm differentiation. Indeed such heterogeneity in Sox2 and T(Bra) levels (as well as other mesodermal and neural transcripts) has been shown by single cell transcriptomic analysis of mouse ES cell-derived cultures resembling our *in vitro*-generated NMPs ^11^. Nevertheless, the clonal-based assay we employed here establishes bipotency of *in vitro*-derived NMPs and reveals the responsiveness of individual cells to environmental signals.

## Data availability

The data referenced by this article are under copyright with the following copyright statement: Copyright: © 2015 Tsakiridis A and Wilson V

Data associated with the article are available under the terms of the Creative Commons Zero "No rights reserved" data waiver (CC0 1.0 Public domain dedication). 

*Figshare:* Supplementary data for ‘Assessing the bipotency of *in vitro*-derived neuromesodermal progenitors’ doi: 10.6084/m9.figshare.1371001 ^19^
