# Supplementary material for: Assessing the bipotency of in vitro-derived neuromesodermal progenitors
Source: F1000Res. 2015 Jul 31;4:100. Originally published 2015 Apr 28. [Version 2] doi: 10.12688/f1000research.6345.2 (PMC4566282; doi:10.12688/f1000research.6345.2)
Supplement: Peer review history

# Peer review: Referee response for version 2

**DOI:** 10.5256/f1000research.7383.r9743

**Published:** 2015-08-03

**Authors:** Patrick P. L. Tam

**Affiliations:** 1 Embryology Unit, Children's Medical Research Institute, Sydney, NSW, Australia

The responses and revisions have addressed the concerns adequately, except for:

3. (d) This is an after-thought argument, as one may not know before the experiments that these markers are not co-expressed in the cells of interest. While it turned out the experimental strategy has not produced any major confounding outcome, it would be prudent to perform the expression analysis using different fluorescent reporters initially.

3. (g) Data of the missing experiment would have enhanced the merit of this study on the differences in clonal types..

I have read this submission. I believe that I have an appropriate level of expertise to confirm that it is of an acceptable scientific standard.

# Peer review: Referee response for version 1

**DOI:** 10.5256/f1000research.6803.r8552

**Published:** 2015-05-12

**Authors:** Kate G. Storey, Laure Verrier

**Affiliations:** 1 Division of Cell and Developmental Biology, College of Life Sciences, University of Dundee, Dundee, UK

Following a study on the differentiation of dual-fated Neuromesodermal Progenitors (NMPs) at the population level, Tsakiridis and Wilson describe here the ability of a single NMP cell to generate both neural and mesodermal derivatives. Using T(Bra)-GFP expressing EpiSCs, the authors performed a clonal analysis of NMP differentiation driven by a FGF2/CHIR regime. They show that single T(Bra)-GFP expressing cells can generate both mesodermal T(Bra)-GFP and neural Sox2 expressing cells in the same clone. This main finding demonstrates the neuromesodermal bipotency of in vitro derived NMP at the single cell level, recapitulating the behaviour of NMPs as identified by retrospective clonal analysis in the mouse embryo in Tzouanacou *et al. *2009.

Overall, the experiments presented are well designed and the results are carefully analyzed. However, the paper would benefit from improvement of specific points.

**Main comments:**

**Methods**

1. The authors should comment on the use of the ROCK inhibitor Y-27632 in their protocol for cell sorting and during subsequent plating at low density.

**Experimental approaches**

1.  Figure 2 describes the co-appearance of distinct T(Bra) ^+^ and Sox2 ^+^ cells from an NMP population treated by FGF2/CHIR as a way to induce simultaneously neural and mesodermal lineages. Why did the authors not perform neural and mesodermal differentiation in parallel using two distinct protocols?
2.  In this study, we are dependent on the previous finding that cells plated at low density give rise mainly (95%) to monoclonal colonies in the time frame of the experiment. It would be reassuring here if the authors had demonstrated that sorted individual T-GFP +ve cells were also Sox2 co-expressing at the time of plating – even if this required immuno-cytochemistry, it would at least establish the proportion of bra/sox2 co-expressing cells in the starting conditions.
3. Does the negative control in Fig3B represent WT EpiSCs treated with the FGF2/CHIR regime, or are they just undifferentiated cells? A good negative control would be to analyse T(Bra)-GFP undifferentiated EpiSCs as they will have the same genetic background as the NMPs analyzed later but won’t express T(Bra). This control should be included as well.
4. Figure 3C shows clones of various sizes in terms of cell number. Can the size of the clone have an impact on cell lineage identity? It would be good to standardize the analysis by looking at the different expression patterns in colonies with comparable cell numbers.
5. Figure 3D and E, the authors comment on the appearance of T(Bra)/Sox2 double negative colonies, and suggest that they are likely to represent further differentiated derivatives. The authors should address this by looking at other neural and mesodermal markers, such as Pax6 or Sox1 and Paraxis. It would be very informative to know what those cells become. Indeed, the suggestion that a higher density of cells is needed for paraxial mesoderm differentiation might indicate that those negative clones are not mesodermal derivatives.

**Minor comments:**

In figure 1, the authors describe the establishment of an NMP population. Using a FGF2/CHIR differentiation protocol, they only obtain around 38% of T(Bra)-GFP/Sox2 coexpression, with maximum 60% of T(Bra)+ cells in the whole population. However, other studies show up to 80% of coexpression in the same conditions. The authors should comment on that.

Figure 2B: the figure legend should indicate “all nuclei” instead of “ell nuclei”.

The legend for figure 3 (D-E) is confusing and should be clarified.

We have read this submission. We believe that we have an appropriate level of expertise to confirm that it is of an acceptable scientific standard, however we have significant reservations, as outlined above.

# Response

**Published:** 2015-07-23

**Authors:** Anestis Tsakiridis

**Affiliations:** University of Edinburgh (Subscriptions), UK

We would like to thank all three reviewers both for their positive comments and constructive suggestions which improved significantly the quality of our manuscript. Our responses below are shown in italics while reviewer comments are in bold.

**Main comments:**

****

**Methods**

1. **The authors should comment on the use of the ROCK inhibitor Y-27632 in their protocol for cell sorting and during subsequent plating at low density.**
*The ROCK inhibitor Y-27632 is a well-established reagent in human embryonic (hES) and epiblast stem cell (EpiSC) culture used to enhance survival of single cells ( Watanabe et al., 2007) and thus we routinely employ it in FACS sorting experiments as a means of counteracting dissociation-induced apoptosis.*

**Experimental approaches**

1. **Figure 2 describes the co-appearance of distinct T(Bra) ^+^ and Sox2 ^+^ cells from an NMP population treated by FGF2/CHIR as a way to induce simultaneously neural and mesodermal lineages. Why did the authors not perform neural and mesodermal differentiation in parallel using two distinct protocols?**
*Clonal analysis is required because pluripotent stem cell differentiation is never 100% efficient at the population level. We thus aimed to assess the ability of single NMP cells to generate both neural and mesodermal derivatives at the same time using conditions promoting the simultaneous emergence of both lineages (i.e. FGF/CHIR treatment). *
2. **In this study, we are dependent on the previous finding that cells plated at low density give rise mainly (95%) to monoclonal colonies in the time frame of the experiment. It would be reassuring here if the authors had demonstrated that sorted individual T-GFP +ve cells were also Sox2 co-expressing at the time of plating – even if this required immuno-cytochemistry, it would at least establish the proportion of bra/sox2 co-expressing cells in the starting conditions.**
*We agree that ideally the extent of T(Bra)/Sox2 co-expression should be assessed at the time of low density plating. However, in our opinion, its determination can only be achieved using a T/Sox2 double reporter cell line. The alternative option suggested by the reviewers involving the use of immunocytochemistry would be technically challenging given that freshly sorted and plated TGFP+ cells require a few hours to attach properly thus precluding antibody staining at the very start of the experiment. *
3. **Does the negative control in Fig3B represent WT EpiSCs treated with the FGF2/CHIR regime, or are they just undifferentiated cells? A good negative control would be to analyse T(Bra)-GFP undifferentiated EpiSCs as they will have the same genetic background as the NMPs analyzed later but won’t express T(Bra). This control should be included as well.**
*The control used in Fig. 3B was indeed differentiated E14tg2a EpiSCs. This is the same genetic background as the TGFP+ ES cells (E14.1, 129/Ola; Fehling et al., 2003) we used to derive the EpiSC line employed in this study. It is not possible to utilize undifferentiated TGFP EpiSCs as a negative control for FACS as they also express significant levels of both T(BRA) protein and the TGFP reporter under self-renewing conditions (i.e. in FGF2 and Activin A) in line with previous reports ( Tsakiridis et al., 2014; Kurek et al., 2015).*
4. **Figure 3C shows clones of various sizes in terms of cell number. Can the size of the clone have an impact on cell lineage identity? It would be good to standardize the analysis by looking at the different expression patterns in colonies with comparable cell numbers.**
*Splitting the data in Fig. 3C-D based on clone size would be a good way to decipher a link between colony cell number and lineage identity acquisition, a possibility which is indeed very interesting.* *However, this type of representation would be more suitable for a larger dataset. Our clone numbers are too small to support any solid conclusions on this issue and we believe that the representation we opted for is the best way to illustrate graphically both parameters (i.e. clone size and lineage composition). We will be happy to incorporate any specific suggestions and or/consider alternative ways of depicting the results.*
5. **Figure 3D and E, the authors comment on the appearance of T(Bra)/Sox2 double negative colonies, and suggest that they are likely to represent further differentiated derivatives. The authors should address this by looking at other neural and mesodermal markers, such as Pax6 or Sox1 and Paraxis. It would be very informative to know what those cells become. Indeed, the suggestion that a higher density of cells is needed for paraxial mesoderm differentiation might indicate that those negative clones are not mesodermal derivatives.**
*This is a good point which deserves further investigation. Our preliminary data indicate that T(Bra) ^-^Sox2 ^-^ colonies are also negative for Sox1. However, a thorough analysis of these clones will require significant effort and is beyond the scope of this short research note. We have added a sentence in the last paragraph of the results section raising the possibility that double negative clones may also comprise differentiated derivatives of single sorted TGFP ^+^Sox2 ^-^ cells which are probably precursors of mesodermal cell types other than paraxial, also emerging upon culture in FGF2/CHIR.*

**Minor comments:**

**In figure 1, the authors describe the establishment of an NMP population. Using a FGF2/CHIR differentiation protocol, they only obtain around 38% of T(Bra)-GFP/Sox2 coexpression, with maximum 60% of T(Bra)+ cells in the whole population. However, other studies show up to 80% of coexpression in the same conditions. The authors should comment on that.**

*Line-to-line variation in terms of differentiation potential is a common phenomenon in pluripotent stem cell cultures (e.g. see Osafune et al., 2008) and in our hands we also observe some variation between different EpiSC lines both in terms of the extent of induction of NMP-like cells upon culture in FGF2/CHIR as well as the timing of their emergence. The lower numbers of T(Bra) ^+^Sox2 ^+^ double positive cells we observed in this study when compared to the high efficiency of induction exhibited by the in vivo derived EpiSC line R04-GFP ( Gouti et al., 2014) is an example of such variation. One interesting possibility is raised by the fact that the T(Bra) reporter line we employ here contains a GFP cassette knocked into the first exon of the T(Bra) gene ( Fehliing et al., 2003). The resulting heterozygosity may lead to lower efficiency of T(Bra) ^+^Sox2 ^+^ cell generation.*

**Figure 2B: the figure legend should indicate “all nuclei” instead of “ell nuclei”.**

*We cannot locate the phrase the reviewers are referring to. The exact figure legend wording in Fig. 2B is “In all cases cell nuclei were visualized using DAPI”.*

**The legend for figure 3 (D-E) is confusing and should be clarified.**

*We have addressed this point in the new version.*

# Peer review: Referee response for version 1

**DOI:** 10.5256/f1000research.6803.r8488

**Published:** 2015-05-12

**Authors:** Jacqueline Deschamps

**Affiliations:** 1 Developmental Biology and Stem Cell Research, Hubrecht Institute, Utrecht, Netherlands

Tsakiridis and Wilson present the evidence that single cells from a neuromesodermal axial progenitor (NMP) population produced *in vitro *from mouse epiblast stem cells (EpiSCs) can give rise to both neural and mesodermal cell descendants, and are therefore truly bipotent.

In earlier work they had demonstrated the existence of NMPs in the posterior aspect of the developing early somite embryos, and characterized these NMPs as being T Brachyury/Sox2 double positive. In more recent experiments they and others succeeded in defining high Wnt and high Fgf signaling conditions to culture EpiSCs into a cell population wherein more than half the cells are NMP-like.  However, it remained to be proven that an individual NMP-like cell expressing both T Brachyury and Sox2 is able to generate neural derivatives (expressing exclusively Sox2), and mesodermal derivatives (expressing exclusively T Brachyury). It is what the authors achieved in this report, by elegantly making use of EpiSCs derived from T Bra Gfp transgenic embryos. By fluorescence activated cell sorting applied to these EpiSCs cultured in NMP-promoting conditions, they could purify the T Bra positive NMPs and analyze their clonal descendants. They clearly obtained neural and mesodermal descendants from single NMP clones, and could demonstrate that the environmental culture conditions influence the bias of individual NMPs to differentiate into cells with a neural versus mesodermal fate.

The experiments are well designed and executed. The methods are well described, and the results are clear, thoroughly analyzed and discussed appropriately. The data in this manuscript convey a clear message that represents an advance in the field.

I have read this submission. I believe that I have an appropriate level of expertise to confirm that it is of an acceptable scientific standard.

# Peer review: Referee response for version 1

**DOI:** 10.5256/f1000research.6803.r8486

**Published:** 2015-05-07

**Authors:** Patrick P. L. Tam, Pierre Osteil

**Affiliations:** 1 Embryology Unit, Children's Medical Research Institute, Sydney, NSW, Australia

This study on the differentiation of single NMP progenitors generated by FGF2/CHIR treatment of EpiSC is a sequel to the previous study on lineage differentiation of these cells at the population level.  Findings of this study are consistent with the assertion that individual T ^+^/Sox2 ^+^ cells are likely to possess dual potential for differentiation into mesoderm (T ^+^ only) and neuroectodermal (Sox2 ^+^ only) cells. This provides the requisite experimental evidence that some cells *in vitro *may have acquire the attributes of the bipotent NMPs, which are presumed to exist in the node-streak interface and the chordoneural hinges in vivo.

Issues to be clarified:

1. Inconsistency of experimental data
  Data shown in Fig 3D indicates that T ^+^ clones contain 2 and 4 cells (the majority) with a few having up to 6 cells.  The example of T ^+^ve clone shown in Fig 3C contains at least 9 cells. This result is not included the dataset of Fig 3D (Day 2 sorted) or E (Day 3 sorted). Were all or only subsets of clones scored for these clonal culture experiments?
2. Quality of the image data
  a. It is difficult to discern the co-expression, or otherwise, of T-GFP and Sox2 in individual cells at the resolution of Fig 1 and 2. 
  b. It appears that cells with mixed gene expression are only found in some colonies (Fig 1), rather than in a salt and pepper manner in every colony. This may require an explanation in the context of clonal development. 
  c. It can be confusing when different colour schemes were used to show the fluorescence results, e.g. T-GFP signals are shown variously in green (Fig 1), white (Fig 2B) and red (Fig 3C), and Sox2 is shown in red (Fig 1) and green (Fig 2B, 3C), rather than red (which is for Tbx6, Fig 2B). Given that the FGF2/CHIR treated cells were sorted based on GFP activity, T-GFP signal should consistently be displayed- in green for all figures.
3. Additional data / information may help:
  a. While it is plausible that the Tbx6 ^+^ cells might be descendents of the mesoderm progenitor, the results do not unequivocally show that they are derived from the T ^+^ve cells.
  b. What is the evidence for that T ^-^/Sox2 ^-^ cells (which also did not expressing Tbx6) were “more differentiated” NMP derivatives?
  c. Absence of Tbx6 cells in low density culture is an intriguing result.  Is there any precedence that the differentiation of Tbx6-expressing cells is dependent on any “paracrine” signals?
  d. Were the Sox2 and Tbx6 immunofluorescence signals captured in emission channel other than for green fluorescence? If not, would the IF results for these two markers be confounded by the T-GFP background?
  e. Which are the examples of two types of mixed clones in the legend (“T ^+^ mixed with Sox2 ^+^” and “T ^+^/Sox ^+^ with T ^-^/Sox2 ^-^“) in the FGF2/CHIR and FGF experiments (Fig. 3D)?
  f. The bottom panel of Fig 3C: The “T ^+^/Sox2 ^+^ mixed with T ^-^/Sox2 ^-^” clone showed no T ^+^/Sox2 ^+^ cells among the four cells in this figure.
  g. Is there any difference in the clonal types between “Sorted at D2-IF at D4” and “Sorted at D3-IF at Day 5 FGF2/CHIR” groups? What is the rationale for testing the effect of an extended culture to Day 3 before sorting, and was there a parallel culture of “FGF2 only” to Day 3?

We have read this submission. We believe that we have an appropriate level of expertise to confirm that it is of an acceptable scientific standard, however we have significant reservations, as outlined above.

# Response

**Published:** 2015-07-23

**Authors:** Anestis Tsakiridis

**Affiliations:** University of Edinburgh (Subscriptions), UK

Our responses below are shown in italics while reviewer comments are in bold.

**Issues to be clarified:**

1. **Inconsistency of experimental data**
**Data shown in Fig 3D indicates that T+ clones contain 2 and 4 cells (the majority) with a few having up to 6 cells.  The example of T+ve clone shown in Fig 3C contains at least 9 cells. This result is not included the dataset of Fig 3D (Day 2 sorted) or E (Day 3 sorted). Were all or only subsets of clones scored for these clonal culture experiments?**
*We wish to thank the reviewers for spotting the mistake. All clone examples shown in Fig. 3C are taken from the culture experiments described in this study and are included in the scoring graphs in Fig. 3D-E. The T(Bra)+Sox2- clone the reviewers are referring to was mistakenly shown in Fig. 3E as consisting of 7 instead of 9 cells. We have rectified the mistake in the new version (3rd clone from the left, depicted in green). We also re-examined all data and found two further errors:*
*(1) a T(Bra)+Sox2+ double positive clone (also shown as an example in Fig. 3C, top row) was accidentally omitted from the top graph in Fig. 3D*
*(2)a 3-cell clone in the same graph was wrongly depicted as containing two Sox2+ cells and a double negative instead of two Sox2+ cells and a T(Bra)+ cell.*
*These changes have now been incorporated into the new figure version and the data is now correct to the best of our knowledge. We would like to apologise for these errors.*
2. **Quality of the image data**
**a. It is difficult to discern the co-expression, or otherwise, of T-GFP and Sox2 in individual cells at the resolution of Fig 1 and 2. **
*We have now included higher magnification images in figures 1 and 2 to address this issue.*
**b. It appears that cells with mixed gene expression are only found in some colonies (Fig 1), rather than in a salt and pepper manner in every colony. This may require an explanation in the context of clonal development.**
*This is an intriguing observation supporting the idea that NMPs segregate separately from other mesodermal/neural precursors emerging simultaneously in culture conditions promoting a late primitive streak-like environment. It is likely to reflect the fact that the starting EpiSC population is heterogeneous in self-renewing conditions, consisting of cells with differential capacity to generate separate distinct lineages. For example we have previously reported that T(Bra)+Sox2+ and T(Bra)+Foxa2+ mesodermal precursors emerge in a mutually exclusive manner after Wnt signaling stimulation of EpiSCs ( Tsakiridis et al., 2014). It may also be true that the generation of NMP-like, T(Bra)+Sox2+ cells is non-synchronous. We have added a sentence in the first paragraph of the results section to comment on this.*
**c. It can be confusing when different colour schemes were used to show the fluorescence results, e.g. T-GFP signals are shown variously in green (Fig 1), white (Fig 2B) and red (Fig 3C), and Sox2 is shown in red (Fig 1) and green (Fig 2B, 3C), rather than red (which is for Tbx6, Fig 2B). Given that the FGF2/CHIR treated cells were sorted based on GFP activity, T-GFP signal should consistently be displayed- in green for all figures.**
*We have adopted the reviewers’ recommendation and in the new figure versions T(Bra) expression appears in green while Sox2 is depicted in the red.*
3. ***Additional data / information may help:***
***a. While it is plausible that the Tbx6+ cells might be descendents of the mesoderm progenitor, the results do not unequivocally show that they are derived from the T+ve cells.***
*We agree that the data presented here do not show directly that Tbx6+ cells are derived from T(Bra)+ cells. To indicate this we added the word “presumed” before the sentence: “maturation of T(Bra)+ cells into Tbx6-positive paraxial mesoderm” at the end of the Results section. However, we believe that our hypothesis is reasonable since it is based on published studies demonstrating that a considerable fraction of paraxial mesoderm arises from T(Bra)+ late primitive streak precursors (e.g. Anderson et al. 2013; Cambray and Wilson, 2007).*
**b. What is the evidence for that T-/Sox2- cells (which also did not expressing Tbx6) were “more differentiated” NMP derivatives?**
*We are interested in characterizing further the T-/Sox2- clones (see also our response to Referees 1 above). We speculate that these are differentiated NMP-derivatives based on the observation that EpiSCs generating NMPs do not (as far as we can tell) lose Sox2 expression during that process and thus its subsequent loss is likely to indicate further differentiation to downstream NMP products. However, in the absence of any solid data on this point we decided to tone down our statements and expand our hypotheses:*
*1) We modified our statement related to the double negative clones (last paragraph of results section) by replacing the phrase “are likely to represent” with “may represent”*
*2) We raised the alternative possibility that these clones may arise from sorted single T(Bra)+ mesodermal precursors.*
**c. Absence of Tbx6 cells in low density culture is an intriguing result.  Is there any precedence that the differentiation of Tbx6-expressing cells is dependent on any “paracrine” signals?**
*Emergence of Tbx6+ cells is likely to depend on FGF signaling ( Ciruna and Rossant, 2001)-we have now incorporated this possibility into the text together with the speculation that Notch-based regulation of Tbx6 transcription may also be critical for cell density effects ( White et al., 2005).We have also shown previously that paracrine Wnt signaling promotes pioneer T(Bra)+ mesodermal precursors in EpiSCs cultured in self-renewal conditions ( Tsakiridis et al., 2014).*
**d. Were the Sox2 and Tbx6 immunofluorescence signals captured in emission channel other than for green fluorescence? If not, would the IF results for these two markers be confounded by the T-GFP background?**
  We find this possibility unlikely especially since the Sox2+, Tbx6+ and TGFP+ expression domains shown in Fig. 2 were in most cases mutually exclusive from each other and very few or no double or triple positive cells (which could potentially represent a background artefact) were observed.
**e. Which are the examples of two types of mixed clones in the legend (“T+ mixed with Sox2+” and “T+/Sox+ with T-/Sox2-“) in the FGF2/CHIR and FGF experiments (Fig. 3D)?**
*The class of clones defined as “T+ mixed with Sox2+” refers to colonies containing T(Bra)+ single positive cells mixed with Sox2+ single positive cells (example shown in the 5th row in Fig. 3C) while the group defined as “T+/Sox+ mixed with T-Sox2- “ describes clones containing T(Bra)+ OR Sox2+ single positive cells mixed with double negatives (example shown in bottom row in Fig. 3C). We would like to apologise for the lack of clarity. We tried to address this issue by making the legend of Fig. 3D-E more detailed.*
**f. The bottom panel of Fig 3C: The “T+/Sox2+ mixed with T-/Sox2-” clone showed no T+/Sox2+ cells among the four cells in this figure.**
*We use the slash (“/”) to indicate “OR” (see our response directly above). Again we apologise for the confusion.*
**g. Is there any difference in the clonal types between “Sorted at D2-IF at D4” and “Sorted at D3-IF at Day 5 FGF2/CHIR” groups? What is the rationale for testing the effect of an extended culture to Day 3 before sorting, and was there a parallel culture of “FGF2 only”to Day 3?**
*We observed a significant number of T(Bra)+Sox2+ cells both at 48 and 72 hrs of culture in FGF2/CHIR and thus we wished to examine whether these two populations are equivalent in their capacity to clonally generate both neural and mesodermal cells. Since the goal was to compare these two populations, we did not carry out an experiment involving a 3 day culture FGF2 prior to sorting and re-plating.*
